# Pediatric Gastric Mucosal Calcinosis

**DOI:** 10.1097/PG9.0000000000000212

**Published:** 2022-07-25

**Authors:** Derek Ngai, Matthias Wolf, Shannon Kelley, Aakash Goyal

**Affiliations:** From the * Division of Pediatric Gastroenterology, Hepatology and Nutrition, Department of Pediatrics, University of Texas Southwestern/Children’s Medical Center, Dallas, TX; † Division of Pediatric Nephrology, Department of Pediatrics, University of Texas Southwestern/Children’s Medical Center, Dallas, TX; ‡ Division of Pathology, Department of Pediatrics, University of Texas Southwestern/Children’s Medical Center, Dallas, TX.

**Keywords:** gastric mucosal calcinosis, calcinosis, pediatric

## Abstract

Gastric mucosal calcinosis (GMC) is the deposition of calcium salts in gastric tissue. We report a case of GMC in a pediatric patient with a prior history of cholelithiasis and nephrolithiasis who presented with abdominal pain and vomiting. Laboratory evaluation did not show any abnormalities. Upper endoscopy revealed numerous gastric nodules; biopsies revealed calcium deposition in the superficial lamina propria. Recognizing GMC may aid with evaluation of a patient with vague gastrointestinal symptoms.

## INTRODUCTION

Gastric mucosal calcinosis (GMC) refers to the deposition of calcium salts in gastric tissue. This finding has been described mainly in the adult population, with only a few reports in children (1–6). This report describes upper endoscopy findings of numerous gastric nodules in a 17-year-old female with abdominal pain and vomiting, who was ultimately diagnosed with GMC despite normal serum electrolytes.

## CASE PRESENTATION

A 17-year-old female presented to clinic with recent onset of abdominal pain and vomiting. Her medical history was significant for a compound heterozygous mutation in the *STAMBP* gene associated with microcephaly, cerebral palsy, intractable seizures, optic atrophy and blindness, and capillary malformation. Other pertinent diagnoses included gastrostomy-tube dependence, cholelithiasis, nephrolithiasis, and immobility. A computed tomography (CT) scan of the abdomen showed unchanged renal calculi without ureteral calculi, a known hiatal hernia, and questionable stranding at the tail of the pancreas, but no calcifications in the stomach.

Prior evaluation of her nephrolithiasis revealed stone composition of 90% Ca-oxalate and 10% Ca-phosphate, consistent with a risk profile of supersaturation for urinary calcium oxalate and calcium phosphate. Hypercalciuria was treated with hydrochlorothiazide and a water/vinegar mixture given daily by her mother. Celery was often included in her blended diet as well. Other urine studies showed a low urinary citrate and low urine volume. Recent bone density study showed increased bone density (forearm +9.3 Standard Deviation Score [SDS], spine +3.6 SDS) compared with three years earlier (forearm +0.1 SDS, spine −0.4 SDS). Previous serum biochemistries, specifically levels of serum calcium, phosphorus, alkaline phosphatase, 25-OH vitamin D, 1, 25-diOH vitamin D, vitamin A, and parathyroid hormone, had always been normal. Endocrinology consult suggested that the increased bone density was likely related to her kidneys and hydrochlorothiazide therapy, especially in the setting of a normal evaluation for hypercalcemia.

**FIGURE 1.**
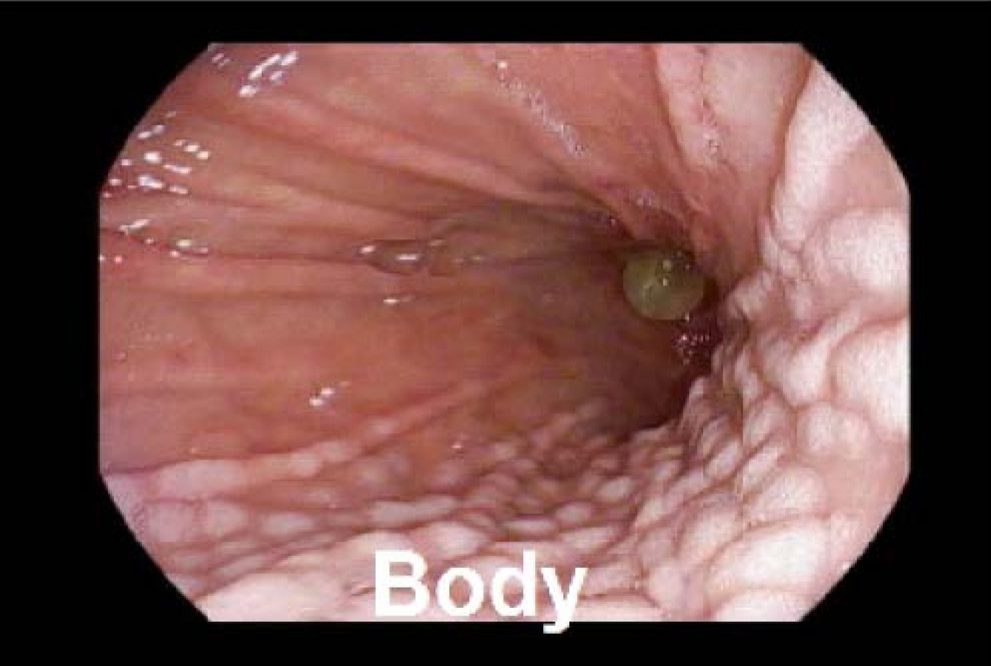
Numerous white nodules and plaques along body of the stomach.

**FIGURE 2.**
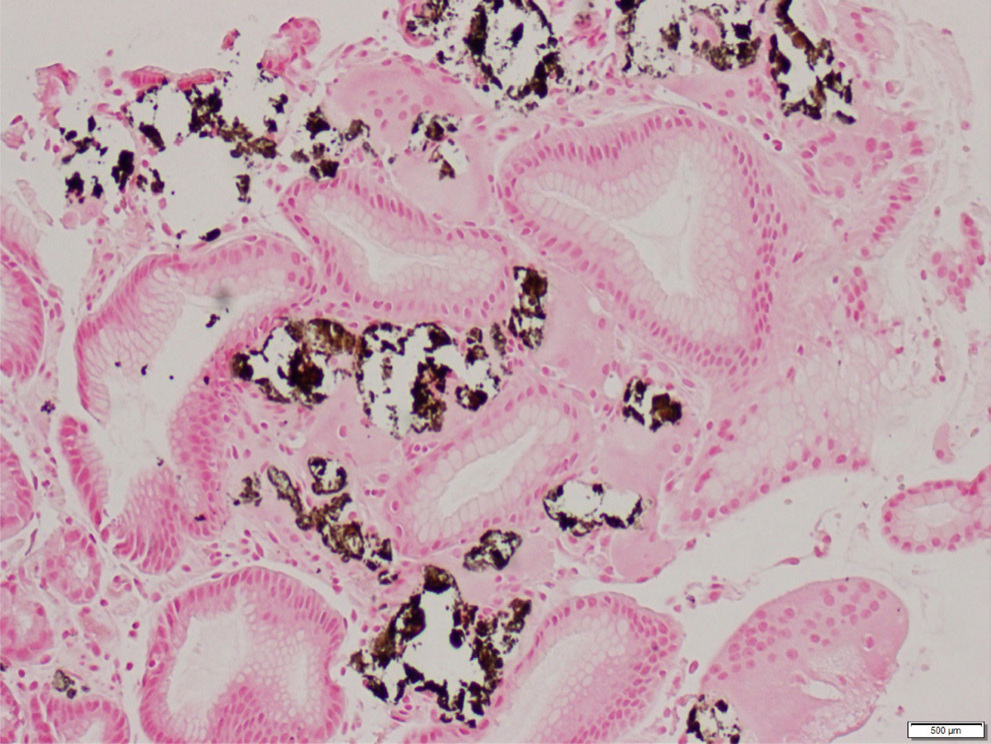
On the Von Kossa stain, calcified material in the lamina propria of the gastric body appears black.

Upper endoscopy, performed for her new abdominal symptoms, revealed white nodules and plaques along the gastric lesser curvature, accompanied by mucosal erythema and friability (Fig. 1). The remainder of the endoscopy appeared normal without an appreciable hiatal hernia. Gastric biopsies revealed oxyntic mucosa with patchy lamina propria inflammation and superficial lamina propria mineralized deposits, determined to be calcium via a Von Kossa stain (Fig. 2), confirming the diagnosis of GMC.

After endoscopy, the hydrochlorothiazide and vinegar therapy were discontinued, and her formula was changed to decrease oxalate, calcium, and sodium intake. Recent follow-up in clinic revealed improved abdominal discomfort and vomiting, and she has remained asymptomatic from her renal stones. With a lack of evidence offering guidance on the management of GMC along with improvement in her clinical symptoms, her mother and care team agreed to not repeat an endoscopy to reevaluate her calcinosis.

## DISCUSSION

GMC is a rare entity described mainly in adults and can be categorized into 3 groups: metastatic, dystrophic, and idiopathic. Metastatic GMC refers to the deposition of calcium salts in normal tissues and is associated with serum electrolyte abnormalities, usually hypercalcemia or hyperphosphatemia secondary to diagnoses such as tumor lysis syndrome and end-stage renal disease (6). Calcifications are more commonly found in the stomach, lungs, and kidneys due to their relative intracellular alkalinity (1,3,7). Dystrophic GMC occurs when calcium salts collect in inflamed, fibrotic, or otherwise altered tissue in the setting of normal serum electrolytes (7). In idiopathic GMC, the cause of the calcification is unclear, and calcium salts are deposited into normal tissues in conjunction with normal biochemical markers (7).

Although metastatic GMC is the most frequently reported type in pediatrics, this patient’s evaluation for increased bone density and gastric calcifications revealed normal serum electrolytes and vitamin levels. Additional studies also showed no evidence for thyroid disease, sarcoid, or diabetes mellitus. Therefore, she fits a diagnosis of dystrophic GMC. It is curious, however, that she was treated with prolonged hydrochlorothiazide therapy. Hydrochlorothiazide improves kidney stones and hypercalciuria by enhancing tubular calcium absorption and can therefore result in hypercalcemia (8). Moreover, vinegar can further reduce gastric pH and enhance gastrointestinal calcium absorption (9). Last, the celery in her blended diet contains high levels of oxalate, which can lead to calcium crystals and renal stones. Despite these theories, her serum calcium values have always been normal. Perhaps, the topical effect of vinegar further decreasing gastric pH may have contributed to the gastric calcifications.

Clinically, GMC is usually asymptomatic, although it has been identified in patients with unexplained dyspepsia or abdominal pain, which may be the case in this patient. GMC is typically an incidental finding seen as white plaques or nodules scattered throughout the stomach. It is important to be aware of this diagnosis, as a variety of causes can lead to GMC. These include electrolyte derangements, atrophic gastritis, gastric ulcer, hypervitaminosis A, organ transplantation, gastric neoplasia, tumor lysis syndrome, and the use of aluminum-containing antacids, citrate-containing blood products, isotretinoin, and sucralfate (1).

## ACKNOWLEDGMENTS

A.G. is guarantor of this article and performed endoscopy, revised manuscript, approved for final submission. D.N. performed endoscopy, drafted and revised manuscript, approved for final submission. M.W. and S.K. revised and contributed to content of manuscript, approved for final submission.
